# Supplementary material for: Estimated standard values of aerobic capacity according to sex and age in a Japanese population: A scoping review
Source: PLoS One. 2023 Sep 15;18(9):e0286936. doi: 10.1371/journal.pone.0286936 (PMC10503723; doi:10.1371/journal.pone.0286936)
Supplement: S1 File — (DOCX) [file pone.0286936.s002.docx]

**Supplemental Material**

**‘Estimated standard values of aerobic capacity according to sex and age in a Japanese population: a scoping review’**

Authors: Hiroshi Akiyama, Daiki Watanabe, Motohiko Miyachi

# **Supporting information**

## ***SUPPLEMENTAL TABLES***

**S1 Table.** Search strategy on the aerobic capacity in the Japanese population.

**S2 Table.** Search strategy on the aerobic capacity in the other populations.

**S3 Table.** Weight-adjusted peak oxygen uptake (VO_2_peak/kg, mL/kg/min) in each age group for Japanese men.

**S4 Table.** Weight-adjusted peak oxygen uptake (VO_2_peak/kg, mL/kg/min) in each age group for Japanese women.

**S5 Table.** Weight-adjusted peak oxygen uptake (VO_2_peak/kg, mL/kg/min) in each age group for men in other populations.

**S6 Table.** Weight-adjusted peak oxygen uptake (VO_2_peak/kg, mL/kg/min) in each age group for women in other populations.

**S7 Table.** Weight-adjusted anaerobic threshold (AT/kg, mL/kg/min) in each age group for Japanese men.

**S8 Table.** Weight-adjusted anaerobic threshold (AT/kg, mL/kg/min) in each age group for Japanese women.

**S9 Table.** Weight-adjusted anaerobic threshold (AT/kg, mL/kg/min) in each age group for men in other populations.

**S10 Table.** Weight-adjusted anaerobic threshold (AT/kg, mL/kg/min) in each age group for women in other populations.

**S11 Table.** The estimated standard values of weight-adjusted peak oxygen uptake (VO_2_peak/kg, mL/kg/min) and anaerobic threshold (AT/kg, mL/kg/min) for men in each age group of other populations.

**S12 Table.** The estimated standard values of weight-adjusted peak oxygen uptake (VO_2_peak/kg, mL/kg/min) and anaerobic threshold (AT/kg, mL/kg/min) for women in each age group of other populations.

**S13 Table.** The equations for estimating the standard values of weight-adjusted peak oxygen uptake (VO_2_peak/kg, mL/kg/min) and anaerobic threshold (AT/kg, mL/kg/min) in each age group of the other populations.

## ***SUPPLEMENTAL FIGURES***

**S1 Fig.** Flow diagram of the article search process for data collected in other populations.

**S2 Fig.** The relationship between age and the estimated standard values of weight-adjusted peak oxygen uptake (VO_2_peak/kg, mL/kg/min) and metabolic equivalents (METs) for Japanese and other populations.

**S3 Fig.** The relationship between age and the estimated standard values of weight-adjusted anaerobic threshold (AT/kg, mL/kg/min) and AT’s metabolic equivalents (METs) for Japanese and other populations.

# ***Supplemental Methods***

We aimed to estimate the standard values of aerobic capacity in the other populations and to compare the estimated standard values of the Japanese with those of other populations. We systematically searched and meta-analysed articles from 1950 to 2022 using PubMed. The data on other populations were systematically searched in umbrella reviews (i.e. examining review articles). The umbrella review is a analysis that integrates multiple reviews and meta-analyses on a specific topic and presents a comprehensive conclusion of the existing evidence. The search terms consisted of relevant terms, such as ‘aerobic capacity’, ‘exercise test’, ‘normal values’, and ‘review’ (S2 Table). Medical Subject Headings were also employed to ensure that potential articles were not missed from the systematic search. The eligibility criteria included studies that reported aerobic capacity by sex and age from countries around the world, including Japan. The aerobic capacity indicators were VO_2_max, VO_2_peak, and AT.

The inclusion criteria were as follows: (1) human studies; (2) studies measuring VO_2_max, VO_2_peak, or AT by exercise tests; (3) studies with study period between 1 January 1950 and 1 January 2022; (4) studies in which data were reported by sex and age; (5) studies in which the exercise mode was cycling or running, because large muscle groups are used and the amount of external load is easy to control; and (6) studies with data from review articles. The exclusion criteria were as follows: (1) studies in which data were not reported by sex and age; (2) studies not in English; (3) studies of individuals with serious diseases, such as angina pectoris, acute myocardial infarction, and chronic obstructive pulmonary disease; (4) studies for which the full text was not available despite requests for articles from the first author and library services; and (5) studies in which data were duplicated (in which case, the study with the more appropriate sample size/reported information was selected).

For primary screening, the papers that potentially contain information on the aerobic capacity of countries around the world, including Japan, by sex and age were selected from titles and abstracts by two independent researchers (H.A. and M.M.). In the secondary screening, the same independent researchers perused the full text of the papers selected in the primary screening and selected those that precisely met the eligibility criteria. The papers handled during selection were managed using Mendeley Desktop (version 1.19.8) between the two researchers. From the papers selected in the secondary screening, data on (1) first author name on Review articles, (2) year of publication, (3) first author name and year of publication on articles included, (4) country, (5) exercise mode, (6) sex, (7) age, and (8) mean values and distribution (SD, standard error, confidence interval [CI]) of VO_2_peak/kg and AT/kg were extracted. The '/kg' here means weight correction, not lean mass. If numerical data were not reported in the included study, we enquired about the numerical data from the corresponding author of the study. If the corresponding author did not respond, numerical data were extracted using WebPlotDigitizer version 4.5 (Ankit Rohatgi, Pacifica, CA, USA) when figures were included in the study. In case of disagreements among the investigators regarding the extracted data, a final decision was made through discussion until a consensus was reached.

Statistical analyses were performed using Microsoft Excel for Windows (version 2206). We calculated VO_2_peak/kg (partly VO_2_max/kg) and AT/kg according to sex and age from the included studies by the simple mean, as the included review articles did not always report the sample size for each age category. Therefore, we did not extract sample size information or use a weighted average.

All statistical analyses were stratified by sex and age group to estimate the standard values of aerobic capacity for the other populations. Age group stratification was calculated using simple mean and 95% CI for each 10-year age group. Scatter plots were created using data on each aerobic capacity and age, and their correlations were examined. Their relationship is influenced by growth and ageing. Then, the slopes and intercepts of the association of age with VO_2_peak/kg and AT/kg for each age category (≤19 years, ≥20 years) were determined using a linear approximation model with the least squares method to consider these influences. The two age categories in the other populations were classified according to the Japanese adult criteria (legal age). Consequently, linear functional equations were developed to predict age-related changes in each aerobic capacity by sex in the other populations. The coefficient of determination (R^2^) was used to assess how well the regression model explains the variables. In addition, considering the effect of the exercise mode on each indicator, we grouped the data into two exercise modes, ‘cycling’ or ‘running’, and calculated the mean and 95% CI for each indicator for each exercise mode.

# ***Supplemental Results***

## ***Accepted article and data extraction for other populations***

We obtained 22 candidate review articles through a systematic search using PubMed. Seven articles were excluded in the primary screening, 8 were excluded in the secondary screening (articles with missing required data = 4, articles comprising unhealthy individuals = 3, articles not written in English = 1), and 7 were accepted. The seven review articles contained 36 original articles. Consequently, 36 articles were included in the meta-analysis (S1 Fig). The total number of individuals was not calculated, because several review articles did not report the number of individuals.

The systematic search extracted and analysed descriptive statistics data of VO_2_peak/kg in other populations from 28 articles on men (20 cycle, 71.4%; 8 run, 28.6%) and 21 articles on women (13 cycle, 61.9%; 6 run, 28.6%; 2 both, 9.5%) (S5 and S6 Tables).

The systematic search extracted and analysed descriptive statistics data of AT/kg in other populations from 6 articles on men (4 cycle, 66.7%; 2 run, 33.3%) and 5 articles on women (4 cycle, 80.0%; 1 run, 20.0%) (S9 and S10 Tables).

## ***Relationship between the estimated standard values of VO_2_peak/kg and age***

S11 and S12 Tables show the estimated standard values of VO_2_peak/kg by sex and age in the other populations. In addition, the relationship between the estimated standard value of VO_2_peak/kg (cycle and run combined) and age is shown in S2 Fig. VO_2_peak/kg was highest during the ages of 10–19 years for men and 0–9 years for women in other populations; thereafter, it declined gradually (S2 Fig, C, F, red circles). To calculate the rate of increase in VO_2_peak/kg with growth during the ages of 10–19 years and the rate of decline in VO_2_peak/kg with ageing after the ages of 20–29 years, linear regression equations with age and VO_2_peak/kg as variables were used (S13 Table).

In the comparison of the estimated standard values of VO_2_peak/kg between Japanese and other populations, Japanese men and women tended to have lower means for all ages (–2.6 mL/kg/min [–6.6%] and –1.7 mL/kg/min [–5.4%], respectively) (S2 Fig, C, F). The difference in range (maximum and minimum values) between Japanese and other populations by sex and age was as follows: men (0.2 to –5.6 mL/kg/min [0.4 to –11.4%], S2 Fig, C) and women (2.0 to –4.0 mL/kg/min [4.8 to –11.0%], S2 Fig, F). In the comparison of the estimated standard values of VO_2_peak/kg by exercise mode, the other population also showed lower levels of VO_2_peak/kg in cycling than in running (S11 and S12 Tables).

## ***Relationship between the estimated standard values of AT/kg and age***

S11 and S12 Tables show the estimated standard values of AT/kg by sex and age in the other populations. In addition, the relationship between the estimated standard value of AT/kg (cycle and run combined) and age is shown in S3 Fig (C, F, red circles). In other populations, AT/kg was highest in the 10–19-year group for both men and women and progressively declined with age (in women, it increased slightly in the 70–79-year group). The linear regression equation between age and AT/kg is shown in S13 Table.

In the comparison of the estimated standard values of AT/kg between Japanese and other populations, Japanese men and women tended to have lower means for all ages (–4.1 mL/kg/min [–19.5%] and –2.1 mL/kg/min [–11.9%], respectively) (S3 Fig, C, F). The difference in range (maximum and minimum values) between Japanese and other populations by sex and age was as follows: men (–3.2 to –5.5 mL/kg/min [–13.3 to -23.1%], S3 Fig, C) and women (–1.1 to –3.6 mL/kg/min [–6.5 to –20.0%], S3 Fig, F).

# **Supplemental data (Tables)**

**S1 Table. Search strategy on the aerobic capacity in the Japanese population.**

| **Systematic search on aerobic capacity in the Japanese population (PubMed)** |
| --- |
| (("maximal oxygen consumption"[Title/Abstract] OR "maximal oxygen uptake"[Title/Abstract] OR "maximum oxygen intake"[Title/Abstract] OR "maximum oxygen uptake"[Title/Abstract] OR "peak oxygen intake"[Title/Abstract] OR "peak oxygen uptake"[Title/Abstract] OR "anaerobic threshold"[Title/Abstract] OR "ventilatory threshold"[Title/Abstract] OR "aerobic fitness"[Title/Abstract] OR "aerobic capacity"[Title/Abstract] OR "aerobic power"[Title/Abstract]) AND ("exercise test"[MeSH Terms] OR "exercise test"[Title/Abstract] OR "ergometry test"[Title/Abstract] OR "ergometry tests"[Title/Abstract] OR "Treadmill test"[Title/Abstract] OR "Treadmill tests"[Title/Abstract] OR "bicycle test"[Title/Abstract] OR "bicycle tests"[Title/Abstract]) AND ("reference values"[MeSH Terms] OR "reference values"[Title/Abstract] OR "normal range"[Title/Abstract] OR "normal ranges"[Title/Abstract] OR norms[Title/Abstract] OR "normative value"[Title/Abstract] OR "normal value"[Title/Abstract] OR "normal values"[Title/Abstract] OR "reference ranges"[Title/Abstract] OR "reference range"[Title/Abstract]) AND (Japan or Japanese) AND ("1950/01/01"[Date - Publication] : "2023/01/01"[Date - Publication])) |
| **Systematic search on aerobic capacity in the Japanese population (Ichushi-Web)** |
| ((全身持久力/TA or 身体持久力/TH or 身体持久力/TA or 最大酸素摂取量/TH or 最大酸素摂取量/TA or 最高酸素摂取量/TA or 嫌気的閾値/TH or 嫌気的閾値/TA or 無酸素性作業閾値/TA or 換気閾値/TH or 換気閾値/TA or 換気性作業閾値/TA) and (運動負荷試験/TH or 運動負荷試験/TA) and (基準値/TH or 基準値/TA) and (DT=1950:2023)) |

**S2 Table. Search strategy on the aerobic capacity in the other populations.**

| **Systematic search on aerobic capacity in the other populations (PubMed)** |
| --- |
| (("maximal oxygen consumption"[Title/Abstract] OR "maximal oxygen uptake"[Title/Abstract] OR "maximum oxygen intake"[Title/Abstract] OR "maximum oxygen uptake"[Title/Abstract] OR "peak oxygen intake"[Title/Abstract] OR "peak oxygen uptake"[Title/Abstract] OR "anaerobic threshold"[Title/Abstract] OR "ventilatory threshold"[Title/Abstract] OR "aerobic fitness"[Title/Abstract] OR "aerobic capacity"[Title/Abstract] OR "aerobic power"[Title/Abstract]) AND ("exercise test"[MeSH Terms] OR "exercise test"[Title/Abstract] OR "ergometry test"[Title/Abstract] OR "ergometry tests"[Title/Abstract] OR "Treadmill test"[Title/Abstract] OR "Treadmill tests"[Title/Abstract] OR "bicycle test"[Title/Abstract] OR "bicycle tests"[Title/Abstract]) AND ("reference values"[MeSH Terms] OR "reference values"[Title/Abstract] OR "normal range"[Title/Abstract] OR "normal ranges"[Title/Abstract] OR norms[Title/Abstract] OR "normative value"[Title/Abstract] OR "normal value"[Title/Abstract] OR "normal values"[Title/Abstract] OR "reference ranges"[Title/Abstract] OR "reference range"[Title/Abstract]) AND (“Meta-Analysis”[Publication Type] OR “Systematic Review”[Publication Type] OR Review[Publication Type]) AND ("1950/01/01"[Date - Publication] : "2022/01/01"[Date - Publication])) |

**S3 Table. Weight-adjusted peak oxygen uptake (VO_2_peak/kg, mL/kg/min) in each age group for Japanese men.**

| **Articles** | **Mode** | ***n*** | **4-9** | **10-19** | **20-29** | **30-39** | **40-49** | **50-59** | **60-69** | **70-79** |
| --- | --- | --- | --- | --- | --- | --- | --- | --- | --- | --- |
|  |  |  | **VO_2_peak/kg (mL/kg/min)** | | | | | | | |
|  |  |  | **Mean** | **Mean** | **Mean** | **Mean** | **Mean** | **Mean** | **Mean** | **Mean** |
| Yamaji and Miyashita (1977) | Cycle | 77 | - | 42.8 | - | - | - | - | - | - |
| Ichikawa & Miyashita (1980) | Cycle | 453 | 47.5 | 49.6 | 49.6 | 38.5 | 36.7 | 35.3 | 30.5 | - |
| Okita (1984) | Cycle | 116 | 50.5 | 54.2 | - | - | - | - | - | - |
| Tamiya (1991) | Cycle | 70 | - | 62.3 | 47.9 | - | - | - | - | - |
| Murayama, JCJ (1992) | Cycle | 173 | - | - | 33.5 | 29.7 | 27.4 | 25.9 | 29.5 | - |
| Ichihara et al (1994) | Cycle | 238 | - | - | - | - | 31.6 | 29.9 | 27.2 | - |
| Kono et al (1997) | Cycle | 37351 | - | - | 42.0 | 39.0 | 37.5 | 36.0 | - | - |
| Tamura (1997) | Cycle | 315 | - | - | 34.0 | 29.7 | 28.0 | 24.7 | 24.3 | - |
| Suto et al (1999) | Cycle | 3102 | - | - | 48.2 | 44.4 | 42.8 | 39.6 | - | - |
| Suzuki et al (2009) | Cycle | 1105 | - | - | 42.9 | 39.2 | 35.5 | 31.7 | 28.0 | 25.2 |
| Kuroda et al (2011) | Cycle | 141 | - | - | 39.2 | 33.2 | 31.9 | 27.7 | 25.3 | - |
| Sawada et al (2012) | Cycle | 8935 | - | - | 40.4 | 36.8 | 34.4 | 33.2 | - | - |
| Ashikaga et al (2021) | Cycle | 274 | - | - | 36.0 | 33.0 | 29.4 | 26.4 | 24.8 | 24.7 |
| Kobayashi et al (1980) | Run | 142 | - | - | 42.7 | 40.6 | 36.3 | 34.0 | 28.8 | 27.1 |
| Ichikawa & Miyashita (1980) | Run | 833 | 43.4 | 44.3 | 42.9 | 30.5 | 32.6 | - | - | - |
| Yoshizawa et al (1983) | Run | 368 | 50.4 | 54.2 | - | - | - | - | - | - |
| Kobayashi et al (1984) | Run | 34 | 47.5 | - | - | - | - | - | - | - |
| Kobayashi et al (1986) | Run | 35 | 46.5 | - | - | - | - | - | - | - |
| Murayama, JCJ (1992) | Run | 109 | - | - | 40.1 | 37.7 | 33.2 | 32.2 | 37.6 | - |
| Ohta et al (1999) | Run | 414 | - | - | - | 34.6 | 30.3 | 27.5 | 24.1 | - |
| Suzuki et al (2009) | Run | 232 | - | - | 56.5 | 50.7 | 44.9 | 39.1 | 33.3 | 28.9 |
| Itoh et al (2013) | Run | 97 | - | - | 52.7 | 40.8 | 38.8 | 32.4 | 29.3 | 25.8 |

***n***, number of subjects; **VO_2_peak/kg**, Weight-adjusted peak oxygen uptake. Data were extracted from 13 articles on cycle and 9 articles on run for Japanese studies (men). Data show the total number of subjects, VO_2_peak/kg in each age category, and exercise mode (Cycle or Run) used for each study. The '/kg' here means weight correction, not lean mass.

**S4 Table. Weight-adjusted peak oxygen uptake (VO_2_peak/kg, mL/kg/min) in each age group for Japanese women.**

| **Articles** | **Mode** | ***n*** | **4-9** | **10-19** | **20-29** | **30-39** | **40-49** | **50-59** | **60-69** | **70-79** |
| --- | --- | --- | --- | --- | --- | --- | --- | --- | --- | --- |
|  |  |  | **VO_2_peak (mL/kg/min)** | | | | | | | |
|  |  |  | **Mean** | **Mean** | **Mean** | **Mean** | **Mean** | **Mean** | **Mean** | **Mean** |
| Ichikawa & Miyashita (1980) | Cycle | 418 | 42.6 | 39.6 | 34.7 | 30.4 | 27.3 | 24.6 | 19.1 | - |
| Okita (1984) | Cycle | 99 | 43.3 | 41.6 | - | - | - | - | - | - |
| Tamiya (1991) | Cycle | 69 | - | 53.8 | 42.4 | - | - | - | - | - |
| Murayama, JCJ (1992) | Cycle | 120 | - | - | 25.7 | 27.3 | 23.6 | 23.8 | 22.7 | - |
| Ichihara et al (1994) | Cycle | 219 | - | - | - | - | 26.0 | 24.3 | 23.1 | - |
| Kono et al (1997) | Cycle | 18170 | - | - | 35.8 | 32.3 | 29.5 | 28.5 | - | - |
| Tamura (1997) | Cycle | 598 | - | - | 26.2 | 25.5 | 23.8 | 21.6 | 19.4 | - |
| Ohta et al (1999) | Cycle | 401 | - | - | - | 27.1 | 24.3 | 22.6 | 20.8 | - |
| Suzuki et al (2009) | Cycle | 2048 | - | - | 33.7 | 31.2 | 28.6 | 26.0 | 23.5 | 21.5 |
| Kuroda et al (2011) | Cycle | 287 | - | - | 29.0 | 28.0 | 24.1 | 23.7 | 22.3 | - |
| Ashikaga et al (2021) | Cycle | 255 | - | - | 29.7 | 29.4 | 26.8 | 24.4 | 22.7 | 20.8 |
| Ichikawa & Miyashita (1980) | Run | 547 | 34.5 | 35.6 | 32.3 | 27.7 | 25.8 | 24.1 | - | - |
| Yoshizawa et al (1983) | Run | 311 | 46.5 | 45.2 | - | - | - | - | - | - |
| Kobayashi et al (1984) | Run | 37 | 42.9 | - | - | - | - | - | - | - |
| Murayama, JCJ (1992) | Run | 109 | - | - | 33.1 | 34.5 | 29.0 | 27.0 | 30.7 | - |
| Suzuki et al (2009) | Run | 319 | - | - | 45.2 | 40.6 | 36.0 | 31.5 | 26.9 | 23.4 |
| Itoh et al (2013) | Run | 93 | - | - | 35.3 | 33.4 | 31.6 | 30.3 | 26.3 | 26.9 |

***n***, number of subjects; **VO_2_peak/kg**, Weight-adjusted peak oxygen uptake. Data were extracted from 11 articles on cycle and 6 articles on run for Japanese studies (women). Data show the total number of subjects, VO_2_peak/kg in each age category, and exercise mode (Cycle or Run) used for each study. The '/kg' here means weight correction, not lean mass.

**S5 Table. Weight-adjusted peak oxygen uptake (VO_2_peak/kg, mL/kg/min) in each age group for men in other populations.**

| **Articles** | **Mode** | **Country** | **8-9** | **10-19** | **20-29** | **30-39** | **40-49** | **50-59** | **60-69** | **70-79** | **80-89** |
| --- | --- | --- | --- | --- | --- | --- | --- | --- | --- | --- | --- |
|  |  |  | **VO_2_peak/kg (mL/kg/min)** | | | | | | | | |
|  |  |  | **Mean** | **Mean** | **Mean** | **Mean** | **Mean** | **Mean** | **Mean** | **Mean** | **Mean** |
| Saltin & Grimby (1968) | Cycle | Sweden | - | - | - | - | 44.0 | 38.0 | 37.0 | - | - |
| Astrand et al (1973) | Cycle | Sweden | - | - | 58.7 | - | 45.3 | - | - | - | - |
| Macek & Vavra (1980) | Cycle | Europe | - | 52.8 | 59.0 | - | - | - | - | - | - |
| Cooper et al (1984) | Cycle | USA | - | 46.0 | - | - | - | - | - | - | - |
| Jones et al (1985) | Cycle | Canada | - | 45.5 | 43.0 | 40.9 | 36.6 | - | 25.5 | - | - |
| Mercier et al (1987) | Cycle | France | - | 49.5 | - | - | - | - | - | - | - |
| Posner et al (1987) | Cycle | USA | - | - | - | 39.9 | - | 31.3 | 27.4 | - | 23.3 |
| Sunnegardh et al (1987) | Cycle | Sweden | 52.7 | 52.2 | - | - | - | - | - | - | - |
| Blackie et al (1989) | Cycle | Canada | - | - | - | - | - | - | 33.0 | - | - |
| Singh et al (1989) | Cycle | Malaysia | - | 56.8 | 49.5 | 39.0 | 32.7 | 28.9 | - | - | - |
| Benefice et al (1990) | Cycle | France | - | 46.3 | - | - | - | - | - | - | - |
| Sjodin & Svedenhag (1992) | Cycle | Sweden | - | 61.1 | - | - | - | - | - | - | - |
| Fairbarn et al (1994) | Cycle | Canada | - | - | 51.7 | 47.4 | 42.3 | 40.3 | 32.9 | 26.3 | - |
| Pothoff et al (1994) | Cycle | Germany | - | - | 41.6 | 34.2 | 32.1 | 27.7 | 25.7 | - | - |
| Prioux et al (1997) | Cycle | France | - | 53.2 | - | - | - | - | - | - | - |
| Ong et al (2002) | Cycle | Singapore | - | - | - | - | 30.7 | - | - | - | - |
| Herdy & Uhlendorf (2011) | Cycle | Brazil | - | 47.4 | 41.9 | 39.0 | 35.6 | 30.0 | - | - | - |
| Funk & Schneider (2012) | Cycle | Germany | - | - | - | - | - | - | 29.2 | 27.4 | - |
| Itoh et al (2013) | Cycle | Japan | - | - | 36.1 | 33.1 | 29.4 | 26.4 | 24.8 | 24.7 | - |
| Bongers et al (2014) | Cycle | Netherlands | 45.9 | 48.4 | - | - | - | - | - | - | - |
| McDonough et al (1970) | Run | USA | - | - | - | - | 39.5 | 36.9 | 30.2 | - | - |
| Bruce et al (1973) | Run | USA | - | - | - | 40.8 | - | 33.8 | - | - | - |
| Nagle et al (1977) | Run | USA | - | 54.8 | - | - | - | - | - | - | - |
| Kobayashi et al (1978) | Run | Japan | - | 49.4 | - | - | - | - | - | - | - |
| Nelson et al (2010) | Run | Canada | - | - | - | 40.6 | 38.4 | 35.2 | 31.6 | - | - |
| Aspenes et al (2011) | Run | Norway | - | - | 54.0 | 48.8 | 46.7 | 42.1 | 38.5 | 34.1 | - |
| Edvardsen et al (2013) | Run | Norway | - | - | 48.6 | 46.2 | 42.7 | 36.8 | 32.4 | 30.1 | - |
| Itoh et al (2013) | Run | Japan | - | - | 52.7 | 40.8 | 38.8 | 32.4 | 29.3 | 25.8 | - |

**VO_2_peak/kg**, Weight-adjusted peak oxygen uptake. Data were extracted from 20 articles on cycle and 8 articles on run for other populations studies (men). Data show the country, VO_2_peak/kg in each age category, and the exercise mode (Cycle or Run) used for each study. The '/kg' here means weight correction, not lean mass.

**S6 Table. Weight-adjusted peak oxygen uptake (VO_2_peak/kg, mL/kg/min) in each age group for women in other populations.**

| **Articles** | **Mode** | **Country** | **8-9** | **10-19** | **20-29** | **30-39** | **40-49** | **50-59** | **60-69** | **70-79** | **80-89** |
| --- | --- | --- | --- | --- | --- | --- | --- | --- | --- | --- | --- |
|  |  |  | **VO_2_peak/kg (mL/kg/min)** | | | | | | | | |
|  |  |  | **Mean** | **Mean** | **Mean** | **Mean** | **Mean** | **Mean** | **Mean** | **Mean** | **Mean** |
| Cooper et al (1984) | Cycle | USA | 38.0 | 34.0 | - | - | - | - | - | - | - |
| Jones et al (1985) | Cycle | Canada | - | 41.2 | 33.1 | 27.6 | 24.3 | - | 20.3 | - | - |
| Posner et al (1987) | Cycle | USA | - | - | - | 32.4 | - | 20.8 | 19.7 | - | 19.2 |
| Sunnegardh et al (1987) | Cycle | Sweden | 45.9 | 43.7 | - | - | - | - | - | - | - |
| Blackie et al (1989) | Cycle | Canada | - | - | - | - | - | - | 24.3 | - | - |
| Docherty et al (1991) | Cycle | England | - | 43.3 | - | - | - | - | - | - | - |
| Fairbarn et al (1994) | Cycle | Canada | - | - | 43.9 | 43.9 | 35.9 | 27.9 | 24.3 | 22.7 | - |
| Pothoff et al (1994) | Cycle | Germany | - | - | 35.4 | 27.9 | 23.6 | 23.2 | 19.9 | - | - |
| Ong et al (2002) | Cycle | Singapore | - | - | - | - | 23.9 | - | - | - | - |
| Herdy & Uhlendorf (2011) | Cycle | Brazil | - | 35.6 | 34.0 | 30.0 | 27.2 | 23.9 | 21.2 | - | - |
| Funk & Schneider (2012) | Cycle | Germany | - | - | - | - | - | - | 21.2 | 22.3 | - |
| Itoh et al (2013) | Cycle | Japan | - | - | 30.0 | 29.7 | 27.0 | 24.4 | 22.7 | 20.8 | - |
| Bongers et al (2014) | Cycle | Netherlands | 39.9 | 41.5 | - | - | - | - | - | - | - |
| Bruce et al (1973) | Run | USA | - | - | 29.8 | - | 25.2 | - | - | - | - |
| Drinkwater et al (1975) | Run | USA | - | 49.3 | 40.8 | 41.7 | 39.6 | 34.9 | 25.9 | - | - |
| Nagle et al (1977) | Run | USA | - | 40.8 | - | - | - | - | - | - | - |
| Aspenes et al (2011) | Run | Norway | - | - | 42.9 | 39.8 | 37.9 | 33.7 | 30.6 | 26.5 | - |
| Edvardsen et al (2013) | Run | Norway | - | - | 40.3 | 37.6 | 33.0 | 30.4 | 28.7 | 23.5 | - |
| Itoh et al (2013) | Run | Japan | - | - | 35.3 | 33.4 | 31.6 | 30.3 | 26.3 | 26.9 | - |
| Astrand et al (1973) | Both | Sweden | - | - | 47.6 | - | 38.4 | - | - | - | - |
| Sidney & Shephard (1977) | Both | Canada | - | - | - | - | - | - | 26.4 | - | - |

**VO_2_peak/kg**, Weight-adjusted peak oxygen uptake. Data were extracted from 13 articles on cycle, 6 articles on run, and two articles on both for other populations studies (women). Data show the country, VO_2_peak/kg in each age category, and the exercise mode (Cycle or Run) used for each study. The '/kg' here means weight correction, not lean mass.

**S7 Table. Weight-adjusted anaerobic threshold (AT/kg, mL/kg/min) in each age group for Japanese men.**

| **Articles** | **Mode** | ***n*** | **4-9** | **10-19** | **20-29** | **30-39** | **40-49** | **50-59** | **60-69** | **70-79** | **80-89** |
| --- | --- | --- | --- | --- | --- | --- | --- | --- | --- | --- | --- |
|  |  |  | **AT/kg (mL/kg/min)** | | | | | | | | |
|  |  |  | **Mean** | **Mean** | **Mean** | **Mean** | **Mean** | **Mean** | **Mean** | **Mean** | **Mean** |
| Murayama, JCJ (1992) | Cycle | 172 | - | - | 19.2 | 16.3 | 16.1 | 16.2 | 17.4 | - | - |
| Ichihara et al (1994) | Cycle | 238 | - | - | - | - | 16.2 | 15.4 | 13.9 | - | - |
| Miura (1996) | Cycle | 305 | - | - | 19.7 | 16.3 | 14.7 | 13.6 | 13.1 | 12.0 | 10.9 |
| Tamura (1997) | Cycle | 315 | - | - | 19.8 | 17.8 | 16.9 | 15.0 | 15.3 | - | - |
| Ohta et al (1999) | Cycle | 391 | - | - | - | 18.2 | 16.5 | 15.3 | 14.4 | - | - |
| Kunitomi et al (2000) | Cycle | 56 | - | - | - | - | 14.0 | 14.0 | 13.3 | - | - |
| Ashikaga et al (2021) | Cycle | 274 | - | - | 18.8 | 17.4 | 16.0 | 15.1 | 14.8 | 15.0 | - |
| Murayama, JCJ (1992) | Run | 108 | - | - | 21.1 | 19.5 | 18.7 | 19.0 | 21.2 | - | - |
| Itoh et al (2013) | Run | 102 | - | - | 28.1 | 22.4 | 22.0 | 19.9 | 19.1 | 18.7 | - |

***n***, number of subjects; **AT/kg**, Weight-adjusted anaerobic threshold. Data were extracted from 7 articles on cycle and 2 on run for Japanese studies (men). Data show the total number of subjects, AT/kg in each age category, and exercise mode (Cycle or Run) used for each study. The '/kg' here means weight correction, not lean mass.

**S8 Table. Weight-adjusted anaerobic threshold (AT/kg, mL/kg/min) in each age group for Japanese women.**

| **Articles** | **Mode** | ***n*** | **4-9** | **10-19** | **20-29** | **30-39** | **40-49** | **50-59** | **60-69** | **70-79** | **80-89** |
| --- | --- | --- | --- | --- | --- | --- | --- | --- | --- | --- | --- |
|  |  |  | **AT/kg (mL/kg/min)** | | | | | | | | |
|  |  |  | **Mean** | **Mean** | **Mean** | **Mean** | **Mean** | **Mean** | **Mean** | **Mean** | **Mean** |
| Murayama, JCJ (1992) | Cycle | 119 | - | - | 15.8 | 16.6 | 15.8 | 16.1 | 15.7 | - | - |
| Ichihara et al (1994) | Cycle | 219 | - | - | - | - | 12.7 | 12.1 | 12.5 | - | - |
| Miura (1996) | Cycle | 305 | - | - | 15.9 | 14.3 | 13.4 | 12.4 | 11.8 | 10.7 | 10.6 |
| Tamura (1997) | Cycle | 598 | - | - | 16.8 | 16.7 | 15.4 | 14.3 | 13.0 | - | - |
| Ohta et al (1999) | Cycle | 374 | - | - | - | 16.3 | 15.4 | 14.2 | 13.9 | - | - |
| Ashikaga et al (2021) | Cycle | 255 | - | - | 16.9 | 16.9 | 15.6 | 15.0 | 14.7 | 13.5 | - |
| Murayama, JCJ (1990) | Run | 109 | - | - | 18.3 | 18.7 | 17.7 | 16.6 | 17.6 | - | - |
| Itoh et al (2013) | Run | 102 | - | - | 20.4 | 19.5 | 19.6 | 19.0 | 16.7 | 18.5 | - |

***n***, number of subjects; **AT/kg**, Weight-adjusted anaerobic threshold. Data were extracted from 6 articles on cycle and 2 on run for Japanese studies (women). Data show the total number of subjects, AT/kg in each age category, and exercise mode (Cycle or Run) used for each study. The '/kg' here means weight correction, not lean mass.

**S9 Table. Weight-adjusted anaerobic threshold (AT/kg, mL/kg/min) in each age group for men in other populations.**

| **Articles** | **Mode** | **Country** | **8-9** | **10-19** | **20-29** | **30-39** | **40-49** | **50-59** | **60-69** | **70-79** |
| --- | --- | --- | --- | --- | --- | --- | --- | --- | --- | --- |
|  |  |  | **AT/kg (mL/kg/min)** | | | | | | | |
|  |  |  | **Mean** | **Mean** | **Mean** | **Mean** | **Mean** | **Mean** | **Mean** | **Mean** |
| Ong et al (2002) | Cycle | Singapore | - | - | - | - | 14.9 | - | - | - |
| Herdy & Uhlendorf (2011) | Cycle | Brazil | - | 30.4 | 25.8 | 24.5 | 22.6 | 19.1 | 15.9 | - |
| Funk & Schneider (2012) | Cycle | Germany | - | - | - | - | - | - | 23.8 | 23.1 |
| Itoh et al (2013) | Cycle | Japan | - | - | 19.1 | 18.4 | 16.4 | 15.4 | 15.2 | 15.3 |
| Nelson et al (2010) | Run | Canada | - | - | - | 29.7 | 28.5 | 27.4 | 24.1 | - |
| Itoh et al (2013) | Run | Japan | - | - | 28.1 | 22.4 | 22.0 | 19.9 | 19.1 | 18.7 |

**AT/kg**, Weight-adjusted anaerobic threshold. Data were extracted from 4 articles on cycle and 2 on run for other populations studies (men). Data show the country, AT/kg in each age category, and exercise mode (Cycle or Run) used for each study. The '/kg' here means weight correction, not lean mass.

**S10 Table. Weight-adjusted anaerobic threshold (AT/kg, mL/kg/min) in each age group for women in other populations.**

| **Articles** | **Mode** | **Country** | **8-9** | **10-19** | **20-29** | **30-39** | **40-49** | **50-59** | **60-69** | **70-79** |
| --- | --- | --- | --- | --- | --- | --- | --- | --- | --- | --- |
|  |  |  | **AT/kg (mL/kg/min)** | | | | | | | |
|  |  |  | **Mean** | **Mean** | **Mean** | **Mean** | **Mean** | **Mean** | **Mean** | **Mean** |
| Ong et al (2002) | Cycle | Singapore | - | - | - | - | 13.7 | - | - | - |
| Herdy & Uhlendorf (2011) | Cycle | Brazil | - | 21.5 | 21.3 | 19.1 | 17.8 | 16.1 | 14.9 | - |
| Funk & Schneider (2012) | Cycle | Germany | - | - | - | - | - | - | 19.7 | 20.8 |
| Itoh et al (2013) | Cycle | Japan | - | - | 17.5 | 17.6 | 16.1 | 15.4 | 15.0 | 14.1 |
| Itoh et al (2013) | Run | Japan | - | - | 20.4 | 19.5 | 19.6 | 19.0 | 16.7 | 18.5 |

**AT/kg**, Weight-adjusted anaerobic threshold. Data were extracted from 4 articles on cycle and 1 article on run for other populations studies (Women). Data show the country, AT/kg in each age category, and exercise mode (Cycle or Run) used for each study. The '/kg' here means weight correction, not lean mass.

**S11 Table.** **The estimated standard values of weight-adjusted peak oxygen uptake (VO_2_peak/kg, mL/kg/min) and anaerobic threshold (AT/kg, mL/kg/min) for men in each age group of other populations.**

| **Men** | **All** | | **Cycle** | | **Run** | |
| --- | --- | --- | --- | --- | --- | --- |
| **Age** | **Mean** | **95% CI** | **Mean** | **95% CI** | **Mean** | **95% CI** |
| **(years)** | **(mL/kg/min)** | | **(mL/kg/min)** | | **(mL/kg/min)** | |
| **VO_2_peak/kg** |  |  |  |  |  |  |
| **8-9** | **49.3** | [48.0, 50.6] | **49.3** | [42.6, 56.0] | **N/A** | N/A |
| **10-19** | **51.0** | [49.7, 52.3] | **50.8** | [47.9, 53.7] | **52.1** | [46.8, 57.4] |
| **20-29** | **48.8** | [46.7, 50.9] | **47.7** | [41.9, 53.5] | **51.8** | [48.6, 55.0] |
| **30-39** | **40.9** | [39.4, 42.4] | **39.1** | [35.6, 42.6] | **43.4** | [40.1, 46.7] |
| **40-49** | **38.2** | [36.4, 40.0] | **36.5** | [32.6, 40.4] | **41.2** | [38.1, 44.3] |
| **50-59** | **33.8** | [32.2, 35.5] | **31.8** | [27.9, 35.7] | **36.2** | [33.5, 38.9] |
| **60-69** | **30.6** | [29.1, 32.1] | **29.4** | [26.3, 32.5] | **32.4** | [29.2, 35.6] |
| **70-79** | **28.1** | [26.8, 29.4] | **26.1** | [24.6, 27.6] | **30.0** | [25.3, 34.7] |
| **80-89** | **23.3** | N/A | **23.3** | N/A | **N/A** | N/A |
| **AT/kg** |  |  |  |  |  |  |
| **8-9** | **N/A** | N/A | **N/A** | N/A | **N/A** | N/A |
| **10-19** | **30.4** | N/A | **30.4** | N/A | **N/A** | N/A |
| **20-29** | **24.3** | [19.0, 29.6] | **22.5** | [17.9, 27.1] | **28.1** | N/A |
| **30-39** | **23.7** | [19.1, 28.3] | **21.5** | [17.3, 25.7] | **26.0** | [18.8, 33.2] |
| **40-49** | **20.9** | [16.1, 25.7] | **18.0** | [14.2, 21.8] | **25.2** | [18.8, 31.6] |
| **50-59** | **20.4** | [15.5, 25.3] | **17.2** | [14.6, 19.8] | **23.6** | [16.3, 31.0] |
| **60-69** | **19.6** | [15.9, 23.3] | **18.3** | [13.9, 22.7] | **21.6** | [16.7, 26.5] |
| **70-79** | **19.0** | [14.6, 23.4] | **19.2** | [13.8, 24.6] | **18.7** | N/A |
| **80-89** | **N/A** | N/A | **N/A** | N/A | **N/A** | N/A |

**VO_2_peak/kg**, weight-adjusted peak oxygen uptake; **AT/kg**, weight-adjusted anaerobic threshold; **95% CI**, 95% confidence interval; **N/A**, data not available for meta-analysis. The '/kg' here means weight correction, not lean mass.

**S12 Table.** **The estimated standard values of weight-adjusted peak oxygen uptake (VO_2_peak/kg, mL/kg/min) and anaerobic threshold (AT/kg, mL/kg/min) for women in each age group of other populations.**

| **Women** | **All** | | **Cycle** | | **Run** | |
| --- | --- | --- | --- | --- | --- | --- |
| **Age** | **Mean** | **95% CI** | **Mean** | **95% CI** | **Mean** | **95% CI** |
| **(years)** | **(mL/kg/min)** | | **(mL/kg/min)** | | **(mL/kg/min)** | |
| **VO_2_peak/kg** |  |  |  |  |  |  |
| **8-9** | **41.3** | [36.6, 45.9] | **41.3** | [36.6, 45.9] | **N/A** | N/A |
| **10-19** | **41.2** | [37.9, 44.5] | **39.9** | [36.6, 43.2] | **45.1** | [36.8, 53.4] |
| **20-29** | **37.6** | [34.1, 41.1] | **35.3** | [30.7, 39.9] | **39.5** | [34.6, 44.4] |
| **30-39** | **34.4** | [30.7, 38.1] | **31.9** | [27.0, 36.8] | **38.1** | [34.6, 41.6] |
| **40-49** | **30.6** | [27.1, 34.1] | **27.0** | [23.3, 30.7] | **34.3** | [29.9, 38.7] |
| **50-59** | **27.7** | [24.5, 30.9] | **24.0** | [21.8, 26.2] | **32.3** | [30.0, 34.6] |
| **60-69** | **24.0** | [22.1, 25.9] | **21.7** | [20.4, 23.0] | **27.6** | [25.8, 29.4] |
| **70-79** | **23.8** | [21.9, 25.7] | **21.9** | [20.8, 23.0] | **25.6** | [23.5, 27.7] |
| **80-89** | **19.2** | N/A | **19.2** | N/A | **N/A** | N/A |
| **AT/kg** |  |  |  |  |  |  |
| **8-9** | **N/A** | N/A | **N/A** | N/A | **N/A** | N/A |
| **10-19** | **21.5** | N/A | **21.5** | N/A | **N/A** | N/A |
| **20-29** | **19.7** | [17.5, 21.9] | **19.4** | [15.7, 23.1] | **20.4** | N/A |
| **30-39** | **18.7** | [17.6, 19.8] | **18.4** | [16.9, 19.9] | **19.5** | N/A |
| **40-49** | **16.8** | [14.3, 19.3] | **15.9** | [13.6, 18.2] | **19.6** | N/A |
| **50-59** | **16.8** | [14.6, 19.0] | **15.7** | [15.0, 16.4] | **19.0** | N/A |
| **60-69** | **16.6** | [14.4, 18.8] | **16.5** | [13.4, 19.6] | **16.7** | N/A |
| **70-79** | **17.8** | [13.9, 21.7] | **17.4** | [10.8, 24.0] | **18.5** | N/A |
| **80-89** | **N/A** | N/A | **N/A** | N/A | **N/A** | N/A |

**VO_2_peak/kg**, weight-adjusted peak oxygen uptake; **AT/kg**, weight-adjusted anaerobic threshold; **95% CI**, 95% confidence interval; **N/A**, data not available for meta-analysis. The '/kg' here means weight correction, not lean mass.

**S13 Table. The equations for estimating the standard values of weight-adjusted peak oxygen uptake (VO_2_peak/kg, mL/kg/min) and anaerobic threshold (AT/kg, mL/kg/min) in each age group of the other populations.**

| **Other populations** | | **Age** | **All** | | **Bicycle** | | **Run** | |
| --- | --- | --- | --- | --- | --- | --- | --- | --- |
|  |  |  | **Equation** | **R^2^** | **Equation** | **R^2^** | **Equation** | **R^2^** |
| **VO_2_peak/kg** |  |  |  |  |  |  |  |  |
| **Men** | World | $\leq$ 19 | Y = 0.41x + 45.2 | 0.07 | Y = 0.39x + 45.6 | 0.06 | Y = 0.88x + 37.8 | 0.17 |
|  | World | $\geq$ 20 | Y = -0.42x + 57.5 | 0.62 | Y = -0.43x + 56.4 | 0.61 | Y = -0.43x + 59.9 | 0.78 |
| **Women** | World | $\leq$ 19 | Y = -0.03x + 40.8 | 0.00 | Y = -0.14x + 42.7 | 0.04 | Y = 1.49x + 18.4 | 0.56 |
|  | World | $\geq$ 20 | Y = -0.33x + 45.9 | 0.57 | Y = -0.31x + 42.2 | 0.63 | Y = -0.29x + 46.8 | 0.66 |
| **AT/kg** |  |  |  |  |  |  |  |  |
| **Men** | World | $\leq$ 19 | N/A | N/A | N/A | N/A | N/A | N/A |
|  | World | $\geq$ 20 | Y = -0.12x + 27.1 | 0.16 | Y = -0.06x + 22.5 | 0.07 | Y = -0.18x + 32.8 | 0.43 |
| **Women** | World | $\leq$ 19 | N/A | N/A | N/A | N/A | N/A | N/A |
|  | World | $\geq$ 20 | Y = -0.05x + 20.1 | 0.12 | Y = -0.04x + 19.2 | 0.07 | Y = -0.05 + 21.5 | 0.60 |

**VO_2_peak/kg**, weight-adjusted peak oxygen uptake; **AT/kg**, weight-adjusted anaerobic threshold; **R^2^**, coefficient of determination; **N/A**, data not available for meta-analysis. Y is VO_2_peak/kg or AT/kg, X is Age. The '/kg' here means weight correction, not lean mass.

# **Supplemental data (Figures)**

**

**

**S1 Fig. Flow diagram of the article search process for data collected in other populations.**

Systematic search aimed to estimate the standard values of aerobic capacity for other populations. Twenty-two review articles were identified in the systematic search, 15 were selected in the evaluation by title and abstract (primary screening), and 7 were included in the evaluation by full-text close reading (secondary screening). ^*^36 articles were eventually extracted from seven review articles and combined in meta-analysis.

**S2 Fig. The relationship between age and the estimated standard values of weight-adjusted peak oxygen uptake (VO_2_peak/kg, mL/kg/min) and metabolic equivalents (METs) for Japanese and other populations.**

VO_2_peak/kg and METs peak (cycle and run combined) at the age of 10 years and then decline progressively after the age of 20–29 years. The estimated standard values of VO_2_peak/kg and METs tend to be lower in Japanese men (0.2 to –5.6 mL/kg/min) and women (2.0 to –4.0 mL/kg/min) than in men and women of other populations. The relationship between age and VO_2_peak /kg and METs is shown for men (**A**) and women (**D**) from Japanese (white circles) and men (**B**) and women (**E**) from other populations (red circles). Triangles indicate age < 20 years, and circles indicate age > 20 years. The estimated standard values of VO_2_peak/kg and METs for each decade for men (**C**) and women (**F**) in Japanese and other populations are shown. The dashed lines show the 95% confidence intervals of the data for Japanese (black) and other populations (red). MET is defined as the weight-adjusted oxygen uptake divided by 3.5 and the number of times more energy is expended in a given activity than in a resting state. The '/kg' here means weight correction, not lean mass.

**S3 Fig. The relationship between age and the estimated standard values of weight-adjusted anaerobic threshold (AT/kg, mL/kg/min) and AT’s metabolic equivalents (METs) for Japanese and other populations.**

AT/kg and AT’s METs (cycle and run combined) at the age of 20 years and then decline progressively after the age of 20–29 years. The estimated standard values for AT/kg and AT’s METs tend to be lower in Japanese men (–3.2 to –5.5 mL/kg/min) and women (–1.1 to –3.6 mL/kg/min) than in men and women of other populations. The relationship between age and AT/kg and AT’s METs is shown for men (**A**) and women (**D**) from Japanese (white circles) and men (**B**) and women (**E**) from other populations (red circles). Triangles indicate age < 20 years, and circles indicate age > 20 years. The estimated standard values of AT/kg and AT’s METs for each decade for men (**C**) and women (**F**) in Japanese and other populations are shown. The dashed lines show the 95% confidence intervals of the data for Japanese (black) and other populations (red). MET is defined as the weight-adjusted oxygen uptake divided by 3.5 and the number of times more energy is expended in a given activity than in a resting state. The '/kg' here means weight correction, not lean mass.
