# Supplementary material for: Estimated standard values of aerobic capacity according to sex and age in a Japanese population: A scoping review
Source: PLoS One. 2023 Sep 15;18(9):e0286936. doi: 10.1371/journal.pone.0286936 (PMC10503723; doi:10.1371/journal.pone.0286936)
Supplement: Attachment — Submitted filename: Review comments.docx [file pone.0286936.s005.docx]

General comments

The authors performed a meta-analysis to estimate the standard values of VO2peak and AT in the Japanese and other populations stratified by sex and age. The theme of the present study is potentially important. Overall, the statistical analyses and data reporting are appropriate. However, unfortunately the manuscript is not well written. This reviewer has a number of comments to improve the quality of the manuscript.

Specific comments

Page 2, line 29: The words “VO2peak/kg and AT/kg in other populations” suddenly appeared in the latter part of Abstract. The authors should explain beforehand on the aerobic capacity in the other population in the background, purpose, and methods sections.

Page 2, lines 31-32: Readers may misunderstand the conclusion sentence. Some readers may think that the decline in the aerobic capacity is lower (or smaller) in Japanese population than the other population when they read only abstract. Please revise the sentence to prevent the potential readers’ confusion.

Page 3, lines 50-55: To the reviewer’s understanding, epidemiological evidence regarding the association between aerobic capacity and disease risk or mortality risk is absolutely necessary to establish a reference value of aerobic capacity. This reviewer cannot understand the reason why the standard value of the aerobic capacity is absolutely needed to establish the reference value of aerobic capacity. Please explain the reason more clearly and concretely.

Page 4, lines 60-68: The reviewer could not clearly understand the rationales of the primary and secondary aims of the present study. It is even unclear that the authors are trying to explain on the primary or secondary aims in the first paragraph of page 4. The authors should clearly mention the rationales of the primary and secondary aims in this paragraph. The authors should focus on mentioning only the purpose of the present study in the last paragraph of the Introduction section.

Page 4, line 71: General readers who are not familiar with meta-analysis may have no idea about the terms “scoping review” as well as “grey literature search”, or “umbrella review”. Please explain more courteously on them in the Materials and methods section or other appropriate sections.

Page 4, lines: 60-61: The sentence is poorly written, and needs to be revised.

Page 5, lines 94-101: VO2max in the selected articles is directly measured by expired gas during maximal exercise test, or it is indirectly estimated by heart rate response during submaximal exercise test (e.g., using an equation of estimated maximum heart rate 220-age)? It is not clearly written whether the directly or indirectly measured VO2max or both are included (as VO2peak) in the present meta-analysis.

Page 6, 102-110: Are these criteria are the criteria for Japanese population or other population or both? The authors performed a meta-analysis of other populations than Japanese population as a secondary aim (one of the major aims of the present study), but they mentioned “studies not in Japanese” as a criterion (line 106).

Please be explicit not only about the eligibility criteria, but also the search strategy, study selection, data analysis for each of the Japanese population analysis and other population analysis.

Page 6, lines 109-110: The sentence is poorly written, and needs to be revised.

Page 6, lines 113-115: There is no description on the roles of the primary and secondary screening. There is also no explanation on the required conditions that articles pass the primary screening to the secondary screening. Please be explicit about that these primary and secondary screenings are used for Japanese population, other population, or both.

In addition, for example, the authors mentioned that “The following items were extracted: (1) first author’s name, (2) year of publication, (3) sex, (4) age, (5) exercise mode, and (6) means and distributions of VO2peak and AT (SD, standard error, and confidence interval [CI])”. Please be explicit about that this (and other each procedure) is conducted as the primary screening or secondary screening.

Page 7, line 127: VO2peak (or VO2max) is sometimes expressed as / kg lean body mass (LBM). The authors should clearly mention at least once that they used /kg body weight (not LBM) in the Materials and methods section (not only in footnotes of Tables and legends of Figures but also in the Methods section).

Page 7, lines 130-131: The reviewer could not understand the meaning of the sentence. Do you mean that the article (ref. 39) reported VO2peak and AT measured by indirect method? It is known that the VO2peak and AT measured by indirectly method are underestimated when they are compared with VO2peak and AT measured by directly method?

Page 7, lines 132-134: The authors showed VO2peak and AT data by 10-year age group in Tables 1 and 2. However, they suddenly explain on the categorization of ≤19 years or ≥20 years, before they explain about the 10-year age group categorization first. It is also unclear that these data analyses are applied to Japanese population or other population, or both populations. Data analysis section is poorly written and should be drastically revised to prevent readers’ confusion.

Page 7, lines 135-136: The authors mentioned “The slopes and intercepts of the mean and age for each indicator from each study were determined using…”. Is it correct? It should be “The slopes and intercepts of the association of age with VO2peak and AT for each age category (≦19 years, ≧20 years) were determined using…”.

Page 8, line 142: Please be explicit about that these are accepted articles and data extraction for Japanese population, or other population than Japanese, or both.

Page 10, Table 1: The authors may touch the possible reason why the VO2peak data measured by run are lower than VO2peak data measured by cycle in the age groups of 4-9 and 10-19 in the Materials and methods section or Discussion section.

Page 10, Table 1: The percentage of AT in VO2peak is approximately 50% in the age groups of young and middle-aged men (Table 1) and women (Table 2), but the percentage is apparently higher in older men and women (55-61% in Tables 1 and 2). To the reviewer’s knowledge, endurance athletes have a higher percentage of AT in VO2peak (>60%), but not in older people. How do the authors explain this phenomenon?

Page 13, lines 216-220: It is unclear that the VO2peak and AT data shown in S2 and S3 figures are measured by cycle, run, or combined with cycle and run.

Page 16, lines 273-279: Based on the AT data in the other age groups than 70–89-year group, it seems that the decrease in AT in Japanese population compared with other population (shown in S3 figure) is greater than the decrease in VO2peak in the Japanese population compared with other population (shown in S2 figure). How do the authors explain this interesting phenomenon?

Page 18, line 310: The authors stated that “identifying the distribution of aerobic capacity in population may help estimate their health status and develop a health promotion strategy” in Abstract. In addition, they mentioned that “estimated standard values may help set or update more practical reference values for health promotion in the Japanese population” in the conclusion. However, this reviewer could not clearly understand the reason why the estimated standard values may help set or update more practical reference values for health promotion in the Japanese population. Please describe more how the standard values of VO2peak and AT estimated by the present study actually contribute to the development of the reference value of the aerobic capacities for future health promotion, in this Perspectives section or other appropriate sections, more clearly and more concretely.
