# Supplementary material for: Estimated standard values of aerobic capacity according to sex and age in a Japanese population: A scoping review
Source: PLoS One. 2023 Sep 15;18(9):e0286936. doi: 10.1371/journal.pone.0286936 (PMC10503723; doi:10.1371/journal.pone.0286936)
Supplement: Attachment — Submitted filename: Response to Reviewers_20230901.docx [file pone.0286936.s007.docx]

We would like to thank the editor and reviewers for reading the manuscript and providing their constructive comments. Our responses to the reviewers are listed in "Response to Reviewers" file and the corresponding corrections have been made in the "Revised Manuscript with Track Changes". The reviewers' comments (in black) are listed below, followed by our responses (in red).

***Responses to Editor’s comments***

***Comment: Please review your reference list to ensure that it is complete and correct. If you have cited papers that have been retracted, please include the rationale for doing so in the manuscript text, or remove these references and replace them with relevant current references. Any changes to the reference list should be mentioned in the rebuttal letter that accompanies your revised manuscript. If you need to cite a retracted article, indicate the article’s retracted status in the References list and also include a citation and full reference for the retraction notice.***

***Our response:*** We checked the reference list according to the editor's comments. The reference list does not include retracted papers. However, there were some cited references in Japanese papers that did not comply with PLOS ONE's submission guideline and ICMJE sample references. Therefore, we modified these cited references according to these guidelines.

***Responses to Reviewer 1’s comments***

***Comment #1:*** *The authors suitably responded to most of the reviewer’s comments. The reviewer believes that the manuscript has been much improved. However, unfortunately, the authors failed in the revision of the Abstract. An abstract is most important, as is an article title.*

***Our response #1:*** We appreciate the constructive comments of the reviewer and have revised the manuscript accordingly. We revised the abstract, which is as important as the article title. We hope the revised abstract will help readers better understand the subject.

***Comment #2***: *The authors responded, “However, this information has been only briefly provided to avoid confusion among readers regarding the primary purpose of this meta-analysis, which was to obtain a standard value estimated of aerobic capacity in the Japanese population (Page 2, Lines 20, 23-24).” If so, they should clearly mention that the comparison of the estimated standard values of the Japanese with those of other populations was performed “as a secondary or supplementary analysis” in the Abstract. Why don’t they simply express so? A brief description does not necessarily mean a secondary description, and it is just a lack of sufficient explanation. The authors also mention that “This study aimed to estimate standard values of aerobic capacity (peak oxygen uptake [VO2peak]/kg and anaerobic threshold [AT]/kg) for the Japanese population stratified by sex and age using a meta-analysis and to compare them with those of other populations” in Abstract. This sentence may also confuse readers, because readers may misunderstand that comparing aerobic capacity with those of other populations is one of the two primary analyses.*

***Our response #2:*** As per the reviewer’s comment, we recognized that the revised abstracts were not well explained and adequately expressed. To avoid confusion among readers, we have revised that the comparison of estimated standard values between the Japanese and other populations was conducted 'as a supplementary analysis'. The revised sentence is: ‘Moreover, a comparison of the estimated standard values of the Japanese with those of other populations was performed as a supplementary analysis’ (Page 2, Lines 20-21).
